# Identification of a novel immune landscape signature as effective diagnostic markers related to immune cell infiltration in diabetic nephropathy

**DOI:** 10.3389/fimmu.2023.1113212

**Published:** 2023-03-08

**Authors:** Huandi Zhou, Lin Mu, Zhifen Yang, Yonghong Shi

**Affiliations:** ^1^ Department of Pathology, Hebei Medical University, Shijiazhuang, China; ^2^ Hebei Key Laboratory of Kidney Disease, Hebei Medical University, Shijiazhuang, Hebei, China; ^3^ Department of Radiotherapy, The Second Hospital of Hebei Medical University, Shijiazhuang, Hebei, China; ^4^ Department of Nephrology, The Second Hospital of Hebei Medical University, Shijiazhuang, Hebei, China; ^5^ Gynecology and Obstetrics, The Fourth Hospital of Hebei Medical University, Shijiazhuang, Hebei, China

**Keywords:** diabetic nephropathy, renal tubulointerstitial injury, diagnose biomarker, immune cells infiltration, CCR2, CX3CR1, SELP

## Abstract

**Background:**

The study aimed to identify core biomarkers related to diagnosis and immune microenvironment regulation and explore the immune molecular mechanism of diabetic nephropathy (DN) through bioinformatics analysis.

**Methods:**

GSE30529, GSE99325, and GSE104954 were merged with removing batch effects, and different expression genes (DEGs) were screened at a criterion |log2FC| >0.5 and adjusted P <0.05. KEGG, GO, and GSEA analyses were performed. Hub genes were screened by conducting PPI networks and calculating node genes using five algorithms with CytoHubba, followed by LASSO and ROC analysis to accurately identify diagnostic biomarkers. In addition, two different GEO datasets, GSE175759 and GSE47184, and an experiment cohort with 30 controls and 40 DN patients detected by IHC, were used to validate the biomarkers. Moreover, ssGSEA was performed to analyze the immune microenvironment in DN. Wilcoxon test and LASSO regression were used to determine the core immune signatures. The correlation between biomarkers and crucial immune signatures was calculated by Spearman analysis. Finally, cMap was used to explore potential drugs treating renal tubule injury in DN patients.

**Results:**

A total of 509 DEGs, including 338 upregulated and 171 downregulated genes, were screened out. “chemokine signaling pathway” and “cell adhesion molecules” were enriched in both GSEA and KEGG analysis. CCR2, CX3CR1, and SELP, especially for the combination model of the three genes, were identified as core biomarkers with high diagnostic capabilities with striking AUC, sensitivity, and specificity in both merged and validated datasets and IHC validation. Immune infiltration analysis showed a notable infiltration advantage for APC co-stimulation, CD8+ T cells, checkpoint, cytolytic activity, macrophages, MHC class I, and parainflammation in the DN group. In addition, the correlation analysis showed that CCR2, CX3CR1, and SELP were strongly and positively correlated with checkpoint, cytolytic activity, macrophages, MHC class I, and parainflammation in the DN group. Finally, dilazep was screened out as an underlying compound for DN analyzed by CMap.

**Conclusions:**

CCR2, CX3CR1, and SELP are underlying diagnostic biomarkers for DN, especially in their combination. APC co-stimulation, CD8+ T cells, checkpoint, cytolytic activity, macrophages, MHC class I, and parainflammation may participate in the occurrence and development of DN. At last, dilazep may be a promising drug for treating DN.

## 1 Introduction

Diabetic nephropathy (DN), which accounts for about 20%–40% of diabetes mellitus (DM), represents the most frequent and devastating microvascular complications caused by DM and is the leading cause of end-stage renal disease (ESRD) worldwide, especially in developing countries (1). It is characterized by injury to both the renal tubules and glomeruli. DN at the early stage can be reversed after treatment, while DN at the late stage will develop into ESRD. Early diagnosis and intervention might maximize the delay of disease progression, which is particularly important for clinical treatment. Traditionally, DN’s diagnosis depended on the presence of microalbuminuria. But growing evidence shows that many of the DN patients with microalbuminuria can return to normal urine, and only a few patients progress to proteinuria. In addition, in nearly one-third of DN patients with a normal range of albuminuria, a progressive decline in renal function like the glomerular filtration rate (GFR) was found. These indicate that it is not enough to detect proteinuria alone to monitor the incidence and progression of DN (2). Besides, the decline in GFR without microalbuminuria was caused by renal tubular injury (3). Unlike tradition, some studies have shown that the injury of renal tubules and renal interstitium may exist in the early stage of DN and play an important role in disease progression. In the past decade, our understanding of the pathogenesis of DN has expanded from glomerular to tubular pathobiology. Renal tubular injury has been increasingly recognized as an early characteristic of DN. Therefore, the study of relevant biomarkers targeting diabetic tubular injury can reveal the renal structure and dysfunction of patients with diabetes earlier, better monitor the progress of DN, and judge the prognosis (4, 5).

As an inflammation and immune-related disease, immune cells in renal tissues with DN, including resident and infiltrating immune-related cells and types, play a vital role in the occurrence and development of DN. Evidence accumulated from experimental and clinical studies indicates that renal inflammation plays a key role in determining whether renal injury progresses during diabetes. Increasing research reveals that many macrophages, lymphocytes, and mast cells exist in the kidney tissue of DN patients (6), which secrete many inflammatory mediators, cytokines, and oxygen free radicals that can directly or indirectly induce kidney tissue damage and accelerate the process of renal fibrosis. Predominantly, macrophages are one of the main infiltrating leucocytes found in diabetic kidneys and are associated with declining renal function in patients with DN (7). There are high correlations between the aggregation of macrophages and the degree of glomerulosclerosis, proteinuria, SCR, and the presence of renal interstitial fibrosis (8). Following this, T cell recruitment to kidney tissues in diabetic patients was correlated with the development and progression of DN at a degree of function second only to macrophages (9). In addition, there was also growing evidence that even in the early stages of DN, B cells, neutrophils, and DCs accumulated in the glomeruli and interstitium, which played a remarkable regulatory role in the pathogenesis of DN. Significantly, it is of great value to evaluate the contribution of immune cells and explore key genes related to immune cells for clarifying the molecular mechanism underlying DN and developing novel and promising immunotherapeutic targets (9–11).

In this study, gene expression data information from the GEO public database, GSE30529, GSE33925, and GSE104954, was merged to seek DEGs. Two datasets, GSE175759 and GSE47184, were used as validation datasets. After merging, functional analysis was conducted by GO, KEGG, and GSEA, and hub genes were identified by PPI and LASSO regression. Following this, ROC was performed to screen efficient diagnostic biomarkers in DN with a cut-off criterion of AUC >0.8 used both sensitivity and specificity >75%. Next, ROC logistic regression was conducted to explore the predictive value of a combination of screened core biomarkers. Moreover, IHC was used to detect the expression levels of core biomarkers in 30 paracarinoma kidney tissues and 40 kidney tissues of patients with DN. Besides, CMap was used to seek promising compounds for treating renal tubulointerstitial injury in DN patients based on the enriched genes from functional analysis. Furthermore, ssGSEA was performed to calculate the immune-related contribution using three merged microarray datasets. Two algorithms, the Wilcoxon test and LASSO regression, were further applied to determine significant immune signatures with different infiltrates. Together, Spearman’s correlation was also used to analyze the correlation between biomarkers and significantly different infiltrates of immune cells. The findings will provide a new view of diagnostic signatures and immune therapeutic targets for DN.

## 2 Material and methods

### 2.1 Data collection, preprocessing, and differential expression gene screening

**Figure 1:**
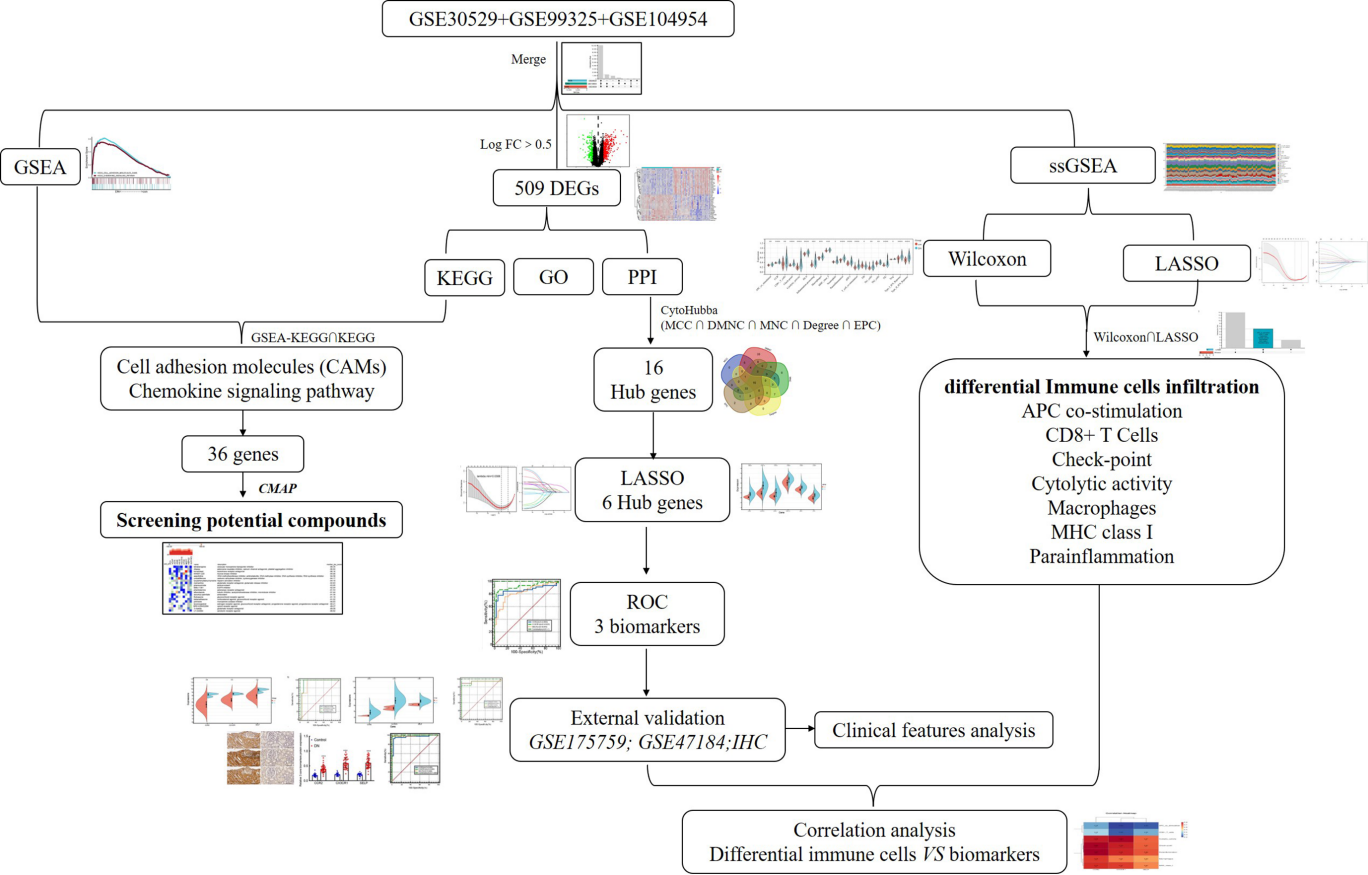
The flow diagram of this study. DEGs, differentially expressed genes; GSEA, gene set enrichment analysis; GO, Gene Ontology; KEGG, Kyoto Encyclopedia of Genes and Genomes; PPI, protein–protein interaction; ROC, receiver operating characteristic.

The flow chart of the study is presented in **Figure 1**. For screening DEGs related to tubulointerstitial injury in patients with DN, three datasets, GSE30529, GSE9325, and GSE104954, were retrieved from the Gene Expression Omnibus (GEO, https://www.ncbi.nlm.nih.gov/geo/) database. In addition, GSE175759 and GSE47184 were also downloaded from GEO for validation. The details are shown in **Table S1**.

After the three test microarray datasets were downloaded from GEO, the probe expression matrixes were converted to gene expression matrixes using the platform annotation file. The values of probe IDs were averaged when genes with ≥1 probe and probes with multiple gene symbols were removed. Then, the three datasets were merged by the “inSilicoMerging” package and batch effects were removed using the method of Johnson et al. (12). After performing batch normalization, the R package “limma” was used to screen DEGs between controls and renal tubulointerstium tissues of DN patients based on |log2FC| >0.5 and adjusted P <0.05. The heat map of DEGs was calculated and mapped using the “Pheatmap” R package.

### 2.2 Gene ontology and kyoto encyclopedia of genes and genomes pathway analysis

The R package “clusterProfiler” was used to perform GO and KEGG enrichment analyses on DEGs, respectively. R software “org.Hs.eg.db” was used for gene ID conversion, and the “goplot” package was used for calculating the Z score. The results were visualized by the R package “ggplot2.” P <0.05 and p.adjust <0.05 were statistically significant.

### 2.3 Gene set enrichment analysis

GSEA was conducted to explore the differentially activated biological pathways between the control and DN groups. The 82 samples in the merged datasets, which belonged to two groups of 37 control samples and 45 DN samples, underwent enrichment analysis using GSEA software (GSEA_4.2.3) on the Java version 8.0 platform. The reference set, gene set c2.cp.kegg.v7.5.symbols.gmt, was obtained from the GSEA official website (http://www.gsea-msigdb.org/gsea/index.jsp) to calculate the enrichment score (ES). It was set at 1,000 permutations, and the gene size was from 5 to 500. A normalized P <0.05 and a false discovery rate (FDR) <0.25 were set as significant thresholds.

### 2.4 Connectivity map analysis

The intersection genes from the intersected pathways between KEGG-DEGs and GSEA-KEGG analysis were uploaded to the query tool of the *Cmap* online platform (https://clue.io/query) to predict promising compounds that may improve tubulointerstitial lesions in DN patients.

### 2.5 Screening hub genes

The STRING platform (http://string-db.org) was used to conduct a protein–protein interaction (PPI) network with medium confidence (score >0.4). Then, the interaction file downloaded from the STRING platform was further analyzed using Cytoscape version 3.9.1 software. The CytoHubba [“CytoHubba: identifying hub objects and subnetworks from complex interactome,” BMC Systems Biology], a Cytoscape software plugin, was used to calculate the node genes using the top five algorithms: MCC, DMNC, MNC, Degree, and EPC. Subsequently, hub genes were screened based on the intersection among the top 60 node genes of each algorithm.

### 2.6 Receiver operating characteristic curve

In public data, ROC analysis was performed by MedCalc software for Windows 20.1.0. The area under the curve (AUC) value >0.8 and both sensitivity and specificity >75% were considered to have better diagnostic effectiveness.

### 2.7 Immunohistochemical staining

A total of 40 patients’ paraffin-embedded samples that were histopathologically and clinically diagnosed as DN were collected at the Second Hospital of Hebei Medical University. A total of 30 samples of paracancerous tissues from normoglycemic renal cancer patients without a history of DN were obtained as normal controls. For the study, patients’ informed consent and approval were obtained from the Ethics Committee of the Second Hospital of Hebei Medical University. The expression of three biomarkers was detected by the IHC method as described in the instructions of ZSGB-BIO (PV-9000, Beijing, China). The immunohistochemical staining score was based on previously published articles (13, 14). The staining estimation was assessed by the ImageJ software (National Institutes of Health).

### 2.8 Single-sample gene set enrichment

A total of 29 immune-related cells and types, representing immune cell species, immune function, and immune-related pathways, were obtained (15). Then a ssGSEA was performed to analyze the enrichment of 29 immune signatures in each sample in the expression file of the merged dataset using the “GSVA” R package.

### 2.9 Screen significant differential immune cells

Based on the infiltration of 22 immune cells in control and DN samples calculated by the CIBERSORT algorithm, two methods, the Wilcoxon test and the least absolute shrinkage and selection operator (LASSO) logistic regression, were performed to screen the differential immune signatures. LASSO was conducted with the “glmnet” package.

### 2.10 Correlation analysis between biomarkers and significant differential immune signatures

The analysis of correlations between biomarkers and significant differential immune signatures was conducted with spearman analysis using the Sangerbox platform, an online tool (http://www.sangerbox.com/tool) (16).

### 2.11 Statistical analysis

Statistical analyses were conducted with R and GraphPad Prism 8.0 (GraphPad Software, Inc.). The correlation between three biomarkers and clinical indicators was performed by GraphPad Prism software (8.0) using Pearson or Spearman analysis based on whether they satisfied the normal distribution or not. The unpaired t test or Mann–Whitney U test was used to evaluate the differences between two groups. ROC was done by MedCalc software (20.1.0) to detect the diagnostic efficiency of biomarkers along with calculated AUCs to evaluate the efficacy of core genes in diagnosing DN. All tests were two-tailed, and the definition of statistical significance is p <0.05.

## 3 Results

### 3.1 Identifying the DEGs involved in tubulointerstitial injury between control and DN samples

**Figure 2:**
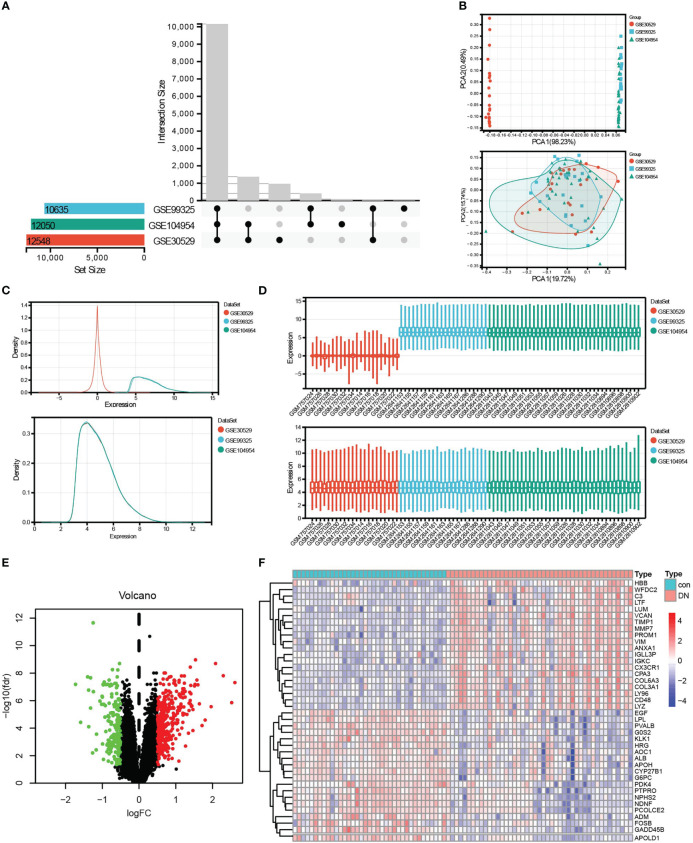
Data preprocessing and DEG screening. **(A)** Upset graph was conducted to obtain the intersection genes in the merge of GSE30529, GSE99325, and GSE104954. Three datasets showed an overlap of 10,635 genes. **(B–D)** The PCA **(B)**, density **(C)**, and box plot **(D)** figures before or after removing batch. **(E)** The final DEGs were visualized by the volcano map, Log2FC >0.5, and adj.P <0.05. **(F)** The top 20 upregulated and top 20 downregulated DEGs were visualized by the heatmap. Red, upregulated differential genes; blue, downregulated differential genes.

According to the research flow chart (**Figure 1**), three datasets, GSE30529, GSE9325, and GSE104954 were downloaded from GEO, and a total of 82 samples (37 controls and 45 DN samples, including 12 controls and 10 DN samples from GSE30529, four controls and 18 DN samples from GSE99325, and 21 controls and 17 DN samples from GSE104954, respectively) containing 10,635 genes (**Figure 2**) were merged to screen DEGs. The PCA (**Figure 2**), density (**Figure 2**), and boxplot (**Figure 2**) diagrams showed that the batch effect of the merged data was better removed. After that, differential expression genes (DEGs) between control samples and DN samples were calculated and screened using the “limma” R package with adjusted P <0.05 and |log2FC| >0.5. A total of 509 DEGs were obtained, which included 338 upregulated genes and 171 downregulated genes. The result was visualized by a volcano map (**Figure 2**), and the top 20 upregulated and top 20 downregulated DEGs were shown in the heatmap (**Figure 2**).

### 3.2 Functional analysis

**Figure 3:**
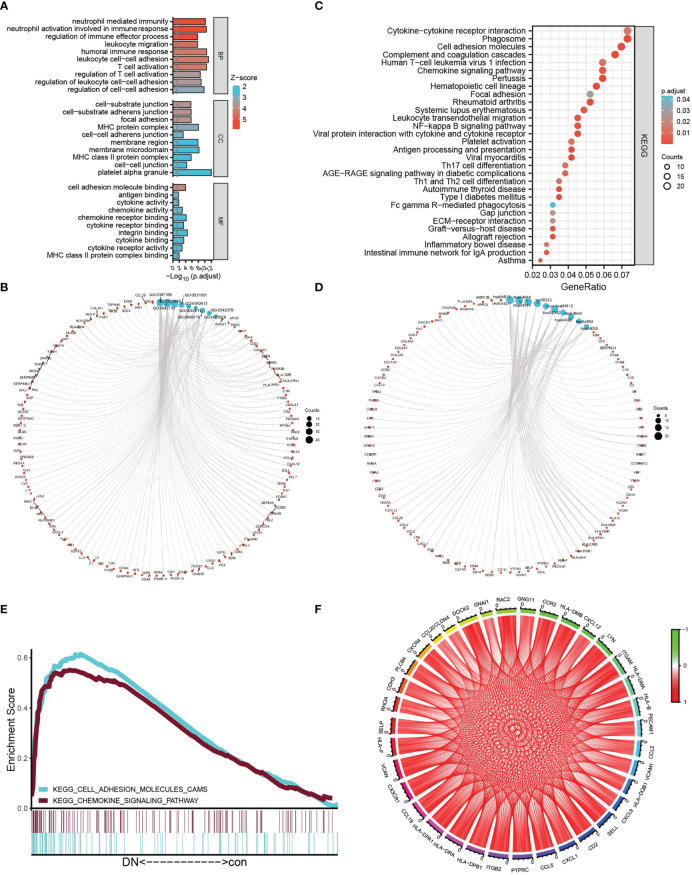
Functional analysis. **(A, B)** The bubble graph **(A)** and circle graph **(B)** of GO analysis for BP, CC, and MF, respectively, based on DEGs. **(C, D)** The bar plot **(C)** and circle charts **(D)** of KEGG analysis based on DEGs. **(E)** Multi-GSEA plot showing the intersection pathways between KEGG analysis of DEGs and GSEA-KEGG-enriched gene sets in the DN group. **(F)** The circle chart shows the correlation of intersection genes between KEGG analysis of DEGs and GSEA–KEGG analysis.

To explore the mechanism related to tubulointerstitial injury in DN patients, after being converted into gene ID, 509 DEGs were analyzed using GO analysis containing BP (biology process), MF (molecular function), CC (cellular component), and KEGG analysis. GO annotation analysis showed a significant correlation with the biological activity of immune cells, for example, “leukocyte cell–cell adhesion,” “T-cell activation,” “neutrophil activation involved in immune response” in BP, “MHC protein complex,” “MHC class II protein complex” in CC, “integrin binding,” “chemokine receptor binding,” “cytokine binding” in MF, and so on (**Figures 3A**). Coincidentally, KEGG analysis of DEGs showed an apparent correlation with immune system and immune disease-related signaling pathways, for example, “complement and coagulation cascades,” “rheumatoid arthritis,” “chemokine signaling pathway,” “autoimmune thyroid disease,” “antigen processing and presentation,” and so on (**Figures 3B**). Moreover, based on the expression profiles of 37 controls and 45 DN samples, GSEA was further employed to explore the gene pathways enriched in different control and DN groups using an annotated gene set (c2.cp.kegg. v7.5.1. symbols) and revealed two intersected pathways with KEGG-DEGs: “chemokine signaling pathway” (NES = 1.48, P = 0.048, FDR = 0.230), and “cell adhesion molecules” (NES = 1.48, P = 0.038, FDR = 0.233), which is shown in **Figure 3**. Subsequently, the intersection genes from the two intersected pathways both in KEGG-DEGs and GSEA-KEGG were calculated, and the correlation of each gene was visualized in the circle graph (**Figure 3**). It was shown that 36 intersection genes had a conspicuous positive correlation.

### 3.3 Identification of hub genes related to renal tubulointerstitial injury in DN group

**Figure 4:**
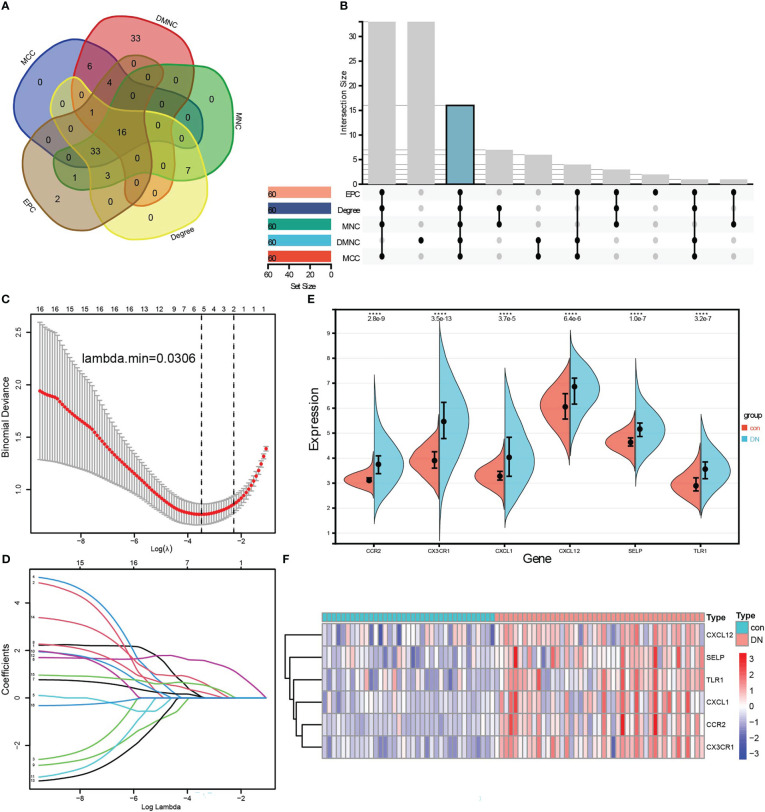
Identification of hub genes related to renal tubulointerstitial injury in DN. **(A, B)** Five algorithms in CytoHubba, a plug-in of Cytoscape software, to screen hub genes. The Venn diagram **(A)** and the Upset graph **(B)** of intersected genes were analyzed by five algorithms: MCC, DMNC, MNC, Degree, and EPC. A total of 16 genes were screened, such as LCP2, CXCL1, CD53, CXCL12, VCAM1, TLR1, CD1C, CSF1R, FCER1G, FCGR2B, CD48, LY86, SELP, CCR2, CX3CR1, and IL10RA. **(C, D)** LASSO regression was conducted to screen further the hub genes, and six genes were screened, such as CCR2, CX3CR1, CXCL1, CXCL12, SELP, and TLR1. **(E)** Wilcoxon test of six hub genes in control and DN samples. **(F)** Six hub genes were visualized by the heatmap.

To identify the hub genes from DEGs, a PPI network was carried out, and the node relationship among genes was obtained from the STRING tool. Then, the score of each node gene was calculated depending on the top five algorithms (MCC, DMNC, MNC, Degree, and EPC) in CytoHubba, a plug-in of Cytoscape software. The top 60 node genes of each algorithm were intersected to screen hub genes, of which a total of 16 genes were selected, such as LCP2, CXCL1, CD53, CXCL12, VCAM1, TLR1, CD1C, CSF1R, FCER1G, FCGR2B, CD48, LY86, SELP, CCR2, CX3CR1, and IL10RA (**Figures 4A**). Furthermore, LASSO regression was conducted to determine the hub genes, and then six genes were screened, such as CCR2, CX3CR1, CXCL1, CXCL12, SELP, and TLR1 (**Figures 4C**). Comparing with control samples, all six genes were upregulated in the DN group in the merged dataset, as shown in the violin chart (**Figure 4**) and the heatmap (**Figure 4**).

### 3.4 Diagnostic effectiveness of six hub genes and validation of screened core genes

**Figure 5:**
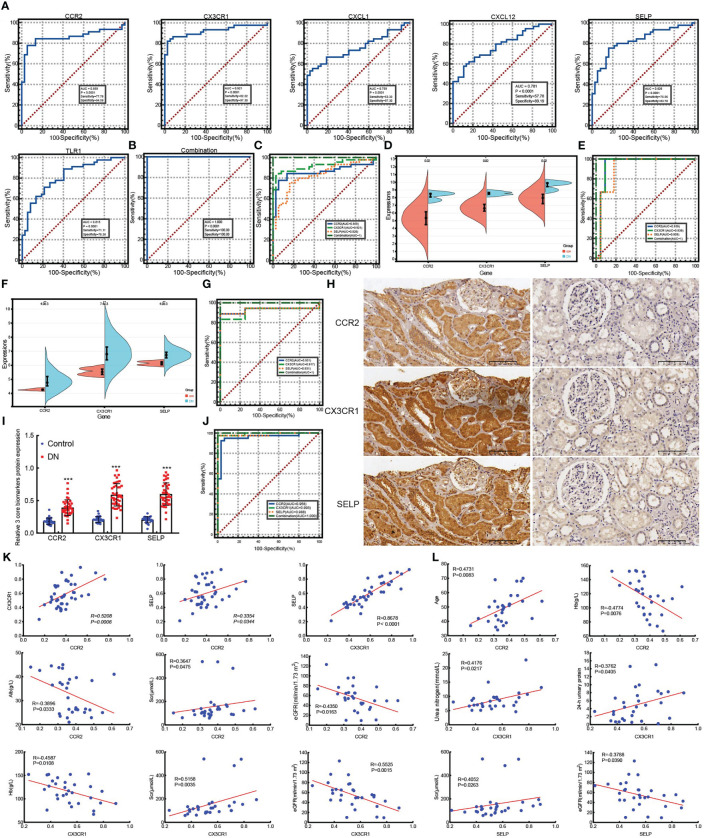
ROC analyze the diagnostic value of six hub genes and external validation of screened biomarkers. **(A)** ROC analyzing the diagnostic value of six hub genes, three core genes of which were screened as biomarkers of DN based on the AUC >0.8, and both sensitivity and specificity >75.00% in merged datasets. **(B)** ROC analysis of the combination model based on three core genes in merged datasets. **(C)** ROC analysis comparing the diagnostic effectiveness among three core genes and the combination model in merged datasets. **(D, E)** The expression validation **(D)** and ROC analysis validation **(E)** of core genes in GSE175759. **(F, G)** The expression validation **(F)** and ROC analysis validation **(G)** of core genes in GSE47184. **(H)** IHC staining examined the expression of three core biomarkers in 30 paracarinoma kidney tissues (right) and 40 kidney tissues of patients with DN (left), (scale bar, 100 μm). **(I)** Expression statistics of IHC staining in 30 paracarinoma kidney tissues and 40 kidney tissues of patients with DN, ***p <0.001 *vs* control. **(J)** ROC analysis comparing the values of three core genes and the combination model in 30 paracarinoma kidney tissues and 40 kidney tissues of patients with DN. **(K)** The correlation among CCR2, CX3CR1, and SELP in 40 kidney tissues of DN patients. **(L)** The correlation between three biomarkers and clinical indicators in 30 kidney tissues of DN patients.

To validate the diagnostic of six hub genes, ROC was conducted to calculate the AUC, specificity, and sensitivity. As shown in **Figure 5**, all six hub genes had an efficient diagnostic value with an AUC >0.75. Especially for CCR2, CX3CR1, and SELP, the three core genes were screened as biomarkers of DN with AUC >0.8, and both sensitivity and specificity >75.00%. Amazingly and meaningfully, the combined AUC of CCR2, CX3CR1, and SELP reached an incredible 1.000 (95% CI 0.956–1.000), with sensitivity = 100% and specificity = 100% (**Figures 5B**). To identify the diagnostic effectiveness of the three biomarkers and their combination, two datasets, GSE175759 and GSE47184, were used to conduct external validation. As shown in **Figures 5D**, each of the three core biomarkers had significantly upregulated expression in DN samples compared to controls in both GSE175759 and GSE47184 (**Figures 5D**). As shown in **Figures 5E**, the AUC values of CCR2, CX3CR1, SELP, and the combination in GSE175759 were 0.939 (95% CI 0.766–0.996), 0.939 (95% CI 0.766–0.996), 0.909 (95% CI 0.725–0.986), and 1.000 (95% CI 0.863–1.000), and the AUC values of CCR2, CX3CR1, SELP, and the combination in GSE47184 were 0.931 (95% CI 0.737–0.995), 0.917 (95% CI 0.718–0.991), 0.931 (95% CI 0.737–0.995), and 1.000 (95% CI 0.846–1.000), respectively (**Figures 5E**).

In addition, to further explore the role of three core biomarkers in DN, protein expression levels detected by IHC were performed on 30 renal cancer paracancerous tissues and 40 DN patients’ biopsy tissues. The clinical characteristics of DN patients are summarized in **Table 1**. According to the degree of 24 h-proteinuria, DN patients were divided into two groups based on the degree of overt proteinuria (n = 16, <3.5 g/24 h) and heavy proteinuria (n =14, >3.5 g/24 h). There was no difference in diabetes history, age, BMI, FBG, SBP, DBP, urea nitrogen, HbA1c, UA, TC, or LDL levels among the two groups. Additionally, the values of 24-h urinary protein, Scr, and TG in the heavy proteinuria group were significantly higher than those in the overt proteinuria group. In contrast, Hb, Alb, and eGFR levels in the heavy proteinuria group were dramatically decreased compared with overt proteinuria (p <0.05) (**Table 1**). As a result, CCR2, CX3CR1, and SELP were strongly stained by IHC in the DN group, especially in the renal tubules (**Figures 5H**). The further ROC confirmed the efficient diagnostic capabilities of all three biomarkers, CCR2, CX3CR1, and SELP. Similarly, the combination model showed the highest diagnostic efficiency for DN (AUC = 1.000, 95% CI 0.949–1.000, sensitivity = 100.00%, specificity = 100.0%, p <0.0001) (**Figure 5**). Furthermore, there were remarkable positive correlations among CCR2, CX3CR1, and SELP (CCR2 vs CX3CR1, R = 0.5208, p = 0.0006; CCR2 vs SELP, R = 0.3354, p = 0.0344; CCR2 vs CX3CR1, R = 0.8678, p <0.0001) in the DN group (**Figure 5**). As shown in **Figure 5**, according to information on clinical parameters, 30 out of 40 DN patients were used to further analyze the correlation. There were substantial positive connections between CCR2 and age (R = 0.4731, p = 0.0083), or Scr (R = 0.3647, p = 0.0475), and a significant negative correlation between CCR2 and Hb (R = −0.4774, p = 0.0076), or Alb (R = −0.3896, p = 0.0333), or eGFR (R = −0.4350, p = 0.0163). We also found that CX3CR1 positively correlated with urea nitrogen (R = 0.4176, p = 0.0217), 24-h urinary protein (R = 0.3762, p = 0.0405), or Scr (R = 0.5158, P = 0.0035), negatively correlated with Hb (R = −0.4587, p = 0.0108), or eGFR (R = −0.5525, p = 0.0015). SELP had a confirmed positive correlation with Scr (R = 0.4052, p = 0.0263) and a negative connection with eGFR (R = −0.3788, p = 0.0390) (**Figure 5**). Therefore, CCR2, CX3CR1, SELP, and their combination were capable of diagnosing control and DN with excellent specificity and sensitivity, especially for the combination.

**Table 1:** Clinical characteristics of the patients with DN.

Parameter	Overt proteinuria (n = 16)	Heavy proteinuria (n = 14)	*P*-value
Diabetes history (years)	7.00 ± 3.95	8.71 ± 6.74	0.3954
Age	47.69 ± 8.48	51.50 ± 11.69	0.3110
BMI (kg/m^2^)	27.08 ± 4.23	27.23 ± 3.52	0.9391
FBG (nmol/L)	8.20 ± 3.48	10.17 ± 5.61	0.2617
SBP (mmHg)	143.56 ± 22.46	152.57 ± 23.58	0.2934
DBP (mmHg)	91.44 ± 13.76	92.36 ± 17.52	0.8733
Urea nitrogen (mmol/L)	7.71 ± 2.42	9.69 ± 4.36	0.1070
24-h urinary protein	3.27 ± 4.61	6.87 ± 1.83	***0.0002***
HbA1c (%)	8.20 ± 1.86	9.53 ± 2.58	0.1131
Hb (g/L)	126.13 ± 20.82	103.86 ± 26.04	***0.0147***
Alb (g/L)	36.81 ± 6.97	28.23 ± 6.64	***0.0121***
Scr (μmol/L)	129.00 ± 113.20	185.86 ± 141.16	***0.0393***
eGFR (ml/min/1.73 m^2^)	66.81 ± 26.15	41.81 ± 18.62	***0.0060***
UA (mmol/L)	387.38 ± 95.61	406.14 ± 90.01	0.5859
TC (mmol/L)	4.71 ± 1.52	5.78 ± 1.59	0.0698
TG (mmol/L)	1.79 ± 0.55	2.70 ± 1.37	***0.0198***
LDL (mmol/L)	3.2 ± 1.37	3.86 ± 1.75	0.2627

m, male; f, female; kg, kilogram; BMI, Body Mass Index; FBG, fasting blood glucose; SBP, systolic blood pressure; DBP, diastolic blood pressure; HbA1c, glycosylated hemoglobin; Alb, serum albumin; Scr, serum creatinine; eGFR: estimated glomerular filtration rate; UA, uric acid; TC, total cholesterol; TG, total triglyceride; LDL, low-density lipoprotein. Bold values represent P <0.05.

### 3.5 Immune-related cells and type infiltration difference in renal tubulointerstitial tissues between control and DN tissues

**Figure 6:**
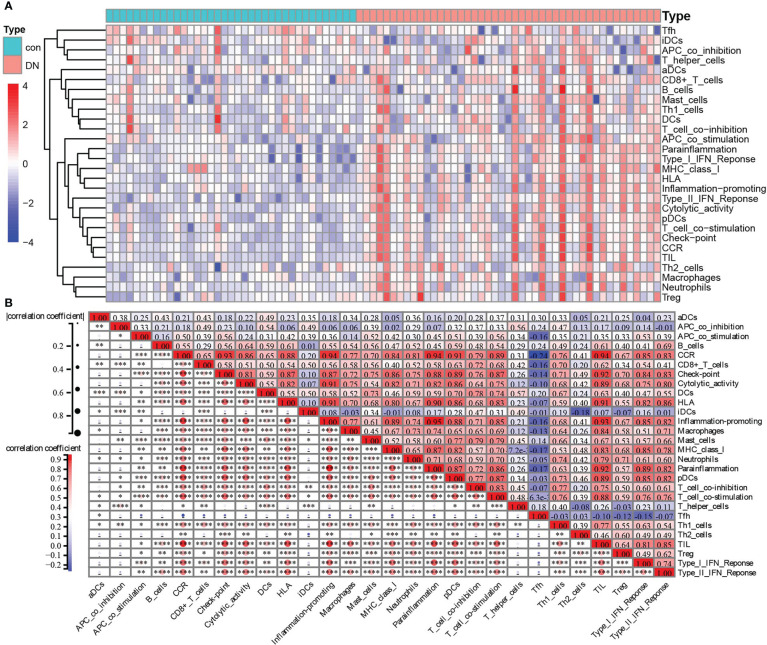
Immune-related cells and types of infiltration difference in renal tubulointerstitial tissues between control and DN tissues. **(A)** The heatmap of the composition of immune signatures in control and DN samples. **(B)** Correlation analyses among the immune signatures calculated by ssGSEA in the DN group: red, positive correlation; blue, negative correlation. *P <0.05, **P <0.01, ***P <0.001, ****P <0.0001.

Since KEGG and GO analysis of DEGs were both enriched to be related to immune cells, the ssGSEA algorithm was applied to evaluate the immune signature infiltration difference so that we could explore the immune microenvironment of DN and further clarify immune signatures closely related to renal tubular injury in patients with diabetes nephropathy. Three datasets, including 37 control and 45 DN samples, were selected to conduct a single-sample gene set enrichment analysis based on a gene set including 29 immune-related cells and types. As shown in **Figure 6**, the heatmap revealed that there was more evident immune infiltration in renal tubular tissue in the DN group than in controls (**Figure 6**). Following this, the correlation of 29 immune-related cells and types was estimated. Preeminently, general positive correlations were observed among immune signatures (**Figure 6**). Especially for some immune-related cells and types, including CCR, checkpoint, cytolytic activity, HLA, inflammation-promoting, macrophages, MHC class I, parainflammation, pDCs, T-cell co-inhibition, T-cell co-stimulation, and TIL, highly positive correlations with a correlation coefficient (cor) >0.8 were found. For example, CCR had a strongly positive correlation with checkpoint, cytolytic activity, HLA, inflammation-promoting, MHC class I, neutrophils, parainflammation, pDCs, T-cell co-stimulation, TIL, type I IFN response, and type II IFN response. Checkpoints were significantly positively related to cytolytic activity, HLA, inflammation-promoting, MHC class I, parainflammation, pDCs, T-cell co-stimulation, TIL, type I IFN response, and type II IFN response. There were positive correlations between cytolytic activity and HLA, or inflammation-promoting, or MHC class I, or parainflammation, or pDCs, or TIL. HLA had positive correlations with inflammation-promoting, parainflammation, pDCs, T-cell co-stimulation, TIL, type I IFN response, and type II IFN response. There were positive correlations between inflammation-promoting MHC class I, parainflammation, pDCs, T-cell co-stimulation, TIL, type I IFN response, and type II IFN response. Macrophages were positively correlated with TIL. MHC class I had a positive correlation with parainflammation, pDCs, TIL, and type I IFN responses. Parainflammation was positively correlated with pDCs, T-cell co-stimulation, TIL, type I IFN response, and type II IFN response. There were positive correlations between pDCs and T-cell co-stimulation, TIL, type I IFN response, and type II IFN response. T-cell co-stimulation was positively correlated with TIL, and T-cell co-inhibition. TIL was positively correlated with type I IFN response, and type II IFN response (**Figure 6**). In sharp contrast, there were declines or reverse correlations among the 29 immune-related cells and types in the control group (**Figure S1**).

**Figure 7:**
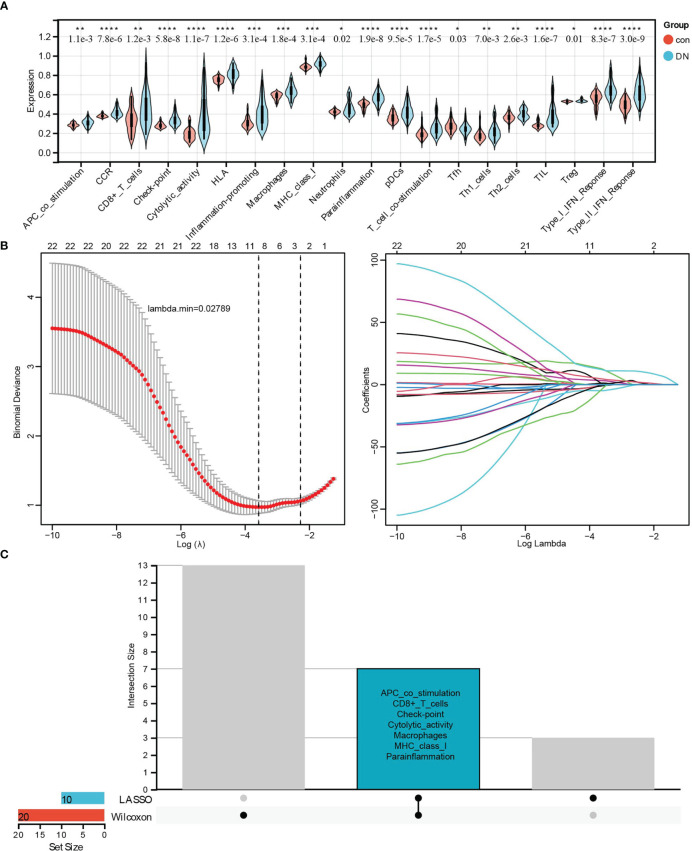
Identifying the significantly different infiltrates of immune-related cells and types related to renal tubulointerstitial injury in DN. **(A)** The violin diagram of 20 types of significant differential immune signatures analyzed by the Wilcoxon test. **(B)** The LASSO regression of immune signatures in control and DN samples. **(C)** The Upset diagram about intersected immune cells between Wilcoxon and LASSO, which showed seven kinds of immune signatures, such as APC co-stimulation, CD8+ T cell, checkpoint, cytolytic activity, macrophages, MHC class I, and proinflammation, were significantly different between control and DN samples. *P < 0.05, **P < 0.01, ***P < 0.001, ****P < 0.0001.

Furthermore, two kinds of algorithms, the Wilcoxon test and LASSO regression, were applied to identify the most related immune signatures. As shown in **Figure 7**, 20 kinds of immune-related cells and types, namely APC co-stimulation, CCR, CD8+ T cells, checkpoint, cytolytic activity, HLA, inflammation-promoting, macrophages, MHC class I, neutrophils, parainflammation, pDCs, T-cell co-stimulation, Tfh, Th1 cells, Th2 cells, TIL, Treg, type I IFN response, and type II IFN response, differed significantly between DN and control group based on the Wilcoxon test (**Figure 7**). In addition, the results from LASSO regression with lambda. min = 0.02789 presented 10 types of immune signatures with p <0.05, such as aDCs, APC co-stimulation, CD8+ T cells, checkpoint, cytolytic activity, iDCs, macrophages, mast cells, MHC class I, and parainflammation (**Figure 7**). After being intersected, seven significantly different types of immune signatures were extracted, namely APC co-stimulation, CD8+ T cells, checkpoint, cytolytic activity, macrophages, MHC class I, and parainflammation (**Figure 7**). Compared with the control group, there were higher infiltrations of APC co-stimulation, CD8+ T cells, checkpoint, cytolytic activity, macrophages, MHC class I, and parainflammation in DN tissues.

### 3.6 Correlation between biomarkers and differential immune signatures in DN patients

**Figure 8:**
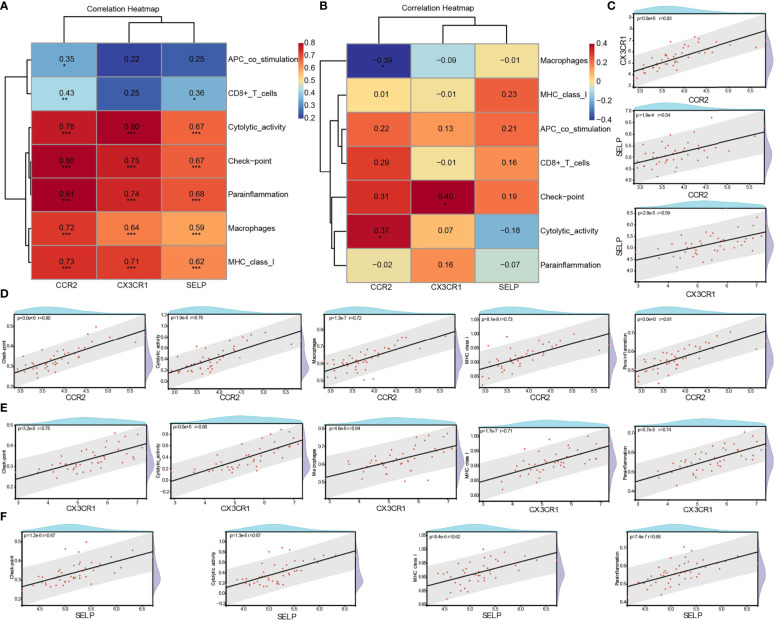
Correlation between biomarkers and differential immune signatures in DN patients. **(A)** Spearman analysis of three core biomarkers and seven significant differential immune signatures in the DN group. **(B)** Spearman analysis of three core biomarkers and seven significant differential immune signatures in the control group. **(C)** The correlation among CCR2, CX3CR1, and SELP in the DN group. **(D–F)** Significant and strong positive correlation between biomarkers and immune signatures. R >0.6 & P <0.05. DN, diabetic nephropathy; *P <0.05, **P< 0.01 ***p <0.001.

The correlation between three core biomarkers (CCR2, CX3CR1, and SELP) and seven differential immune-related signatures (APC co-stimulation, CD8+ T cells, checkpoint, cytolytic activity, macrophages, MHC class I, and parainflammation) was analyzed by Spearman. There were general positive correlations between three biomarkers and seven immune signatures in the DN group (**Figure 8**), and weakened or opposite correlations among them in controls (**Figure 8**). Besides, a strong positive correlation among these three biomarkers was also observed. CCR2 was positively correlated with CX3CR1 (cor = 0.83, p <0.0001) and SELP (cor = 0.54, p <0.0001), and CX3CR1 had a positive correlation with SELP (cor = 0.59, p <0.0001) (**Figure 8**). It was resulting that CCR2 had a significantly positive correlation with all of these seven types of immune signatures, especially for checkpoint (cor = 0.80, p <0.0001), cytolytic activity (cor = 0.76, p <0.0001), macrophages (cor = 0.72, p <0.0001), MHC class I (cor = 0.73, p <0.0001), and parainflammation (cor = 0.81, p <0.0001) (**Figure 8**). CX3CR1 significantly and positively correlated with five out of these seven types of immune signatures, such as checkpoint (cor = 0.75, p <0.0001), cytolytic activity (cor = 0.80, p <0.0001), macrophages (cor = 0.64, p <0.0001), MHC class I (cor = 0.71, p <0.0001), and parainflammation (cor = 0.74, p <0.0001) (**Figure 8**). SELP had significant positive correlations with six out of these seven kinds of immune-related cells and types, especially checkpoint (cor = 0.67, p <0.0001), cytolytic activity (cor = 0.67, p <0.0001), MHC class I (cor = 0.62, p <0.0001), and parainflammation (cor = 0.68, p <0.0001) with high correlations (**Figure 8**).

### 3.7 Exploration of potential compounds to improve diabetic tubulointerstitial injury by CMap analysis

**Figure 9:**
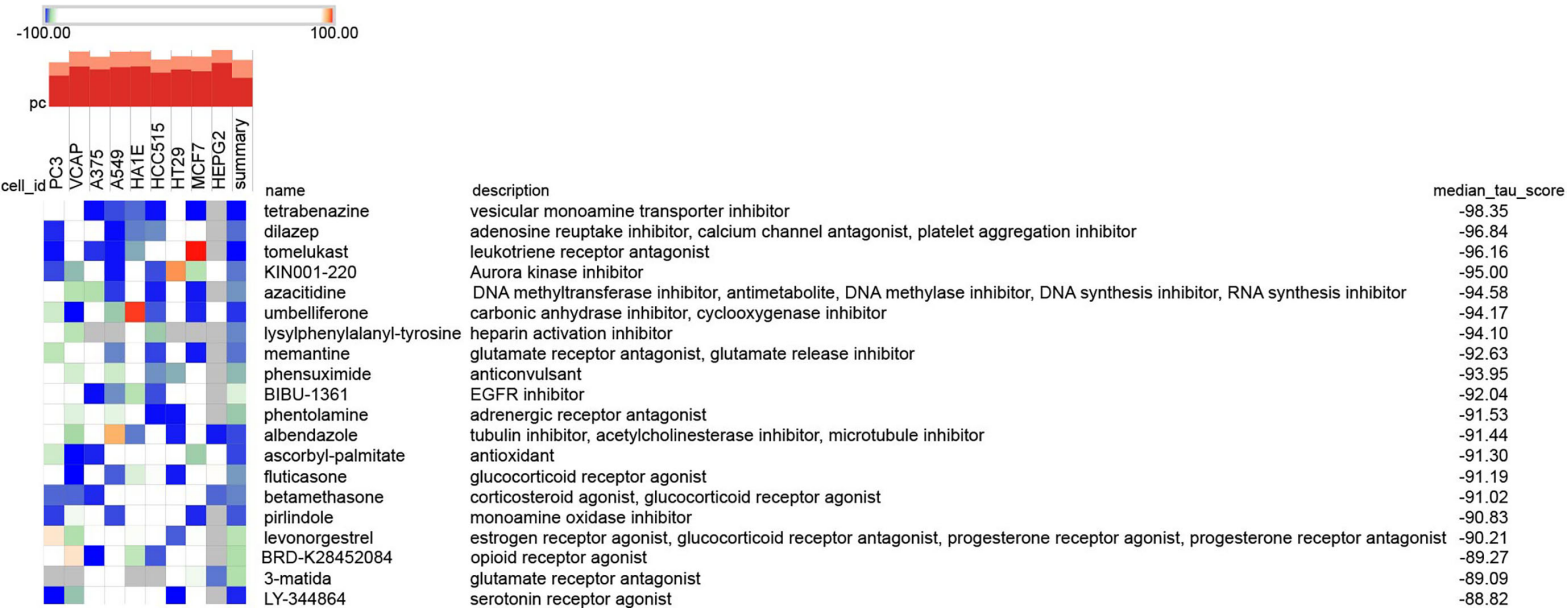
The promising compounds for tubulointerstitial injury in DN analyzed by *CMap.* The 36 intersection genes in two intersected pathways between KEGG-DEGs and GSEA-KEGG analyses were analyzed as potential compounds by the Query tool from the *Cmap* online platform (https://clue.io/query). The top 20 negative compounds were shown in the heat.

To research the promising drugs for treating the tubulointerstitial injury in DN patients, 36 intersected genes from two intersected pathways were uploaded to CMap, which showed the top 20 negative correlation compounds based on median score in **Figure 9**, indicating that these could be reversing the gene alterations in different cell lines (**Figure 9**). As shown in the heatmap, tetrabenazine (vesicular monoamine transporter inhibitor), dilazep (adenosine reuptake inhibitor, calcium channel antagonist, platelet aggregation inhibitor), tomelukast (leukotriene receptor antagonist), KIN001-220 (Aurora kinase inhibitor), azacytidine (DNA methyltransferase inhibitor, antimetabolite, DNA methylase inhibitor, DNA synthesis inhibitor, RNA synthesis inhibitor), umbelliferone (carbonic anhydrase inhibitor, cyclooxygenase inhibitor), lysylphenylalanyl-tyrosine (heparin activation inhibitor), memantine (glutamate receptor antagonist, glutamate release inhibitor), phensuximide (anticonvulsant), and BIBU-1361 (EGFR inhibitor) ranked in the top 10.

## 4 Discussion

Diabetes mellitus (DM) is an endocrine and metabolic disease that can lead to dysfunction of all organs in the body, of which DN is a highly prevalent and serious chronic microvascular complication in patients with diabetes. About 20%–40% of diabetes can progress to DN (1). It is a leading contributor of DN to kidney failure in developed countries (17, 18). Therefore, early diagnosis and intervention in DN are particularly important. At present, renal biopsy histopathology is still the gold standard for diagnosing DN, but it is traumatic and limited in clinical application. Urinary microalbumin (UmAlb) is a widely used indicator for diagnosing DN. However, about 30%–45% of type 2 diabetes patients were observed to have reduced GFR with no increase in UmAlb (>30 mg/g) (19). In addition, the common comorbidities of T2DM, such as hypertension or obesity, may also damage the glomerular filtration barrier, leading to an increase in UmAlb, suggesting that the sensitivity and specificity of UmAlb in diagnosing DN are insufficient. So far, it has been hard for us to accurately predict which one with diabetes will develop DN. Consequently, searching for novel and capable biomarkers for diagnosing DN is of great significance for early treatment and improving the prognosis of patients. In the development of DN, tubulointerstitial injury plays a pivotal role, even prior to glomerular injury. It is characterized by renal tubular atrophy and tubulointerstitial fibrosis, which are considered the main pathological features of renal dysfunction in patients with DN. Tubulointerstitial injuries are more appropriate and useful in predicting renal disease status in DN patients than glomerular or vascular damage. Momentously, more attention should be given to the biomarkers of renal tubule lesions, which are of great value to the diagnosis and treatment of patients in the early stages of DN.

Growing studies showed that biomarkers based on renal tubules can early reveal the renal structure and dysfunction of diabetes patients and better monitor the progress of DN and judge the prognosis, such as kidney injury molecule-1(KIM-1), β2-microglobulin (B2M), N-acetyl-β-D-glucosaminidase (NAG), osteopontin (OPN), etc. KIM-1, a transmembrane glycoprotein of proximal tubular epithelial cells of the kidney, cannot be detected when the kidney is structurally or functionally normal, but it can be significantly upregulated with tubular damage. Therefore, KIM-1 can be used as a potential biomarker for proximal tubule injury (20). B2M is a small subunit of major histocompatibility class I molecules that exists in all nucleated cells. B2M is fully filtered at the glomerulus and then almost completely reabsorbed in the proximal tubule (20). NAG is a hydrolase widely distributed in organs. NAG, with a molecular weight of 130,000, is not easy to filter by the glomerulus. However, when the renal convoluted tubules are damaged, lysozyme will release a large amount of NAG, resulting in a significant increase in NAG in urine (21). OPN, one of the proinflammatory cytokines, was observed to be upregulated in the kidneys of diabetic animals and patients with nephropathy (22). The association of these biomarkers with DN has been found in many studies. There were different capabilities to detect DKD. One study showed an AUC of 0.68 for KIM-1 in diagnosing moderately increased albuminuria (23). B2M had moderate to low AUCs of 0.58 (24), 0.652 (25), and 0.792 (26) to predict early DN in three separate studies. According to the results of two studies, NAG exhibited modest predictive ability for assessing renal tubulointerstitial injury with AUCs of 0.636 (24) and 0.783 (27). OPN exhibited qualified performance with AUCs of 0.692 (28) and 0.73 (29), and did not associate with albuminuria levels, p >0.05 (30). Recently, with the development and widespread application of the human genome project, technologies such as transcriptomics, proteomics, and metabolomics have emerged in succession. Bioinformatic analysis has been a new way to identify novel genes and early diagnosis/prognosis biomarkers for many diseases (4, 31, 32). Liu et al. (33) found that LUM, ELN, and FMOD had the potential abilities to diagnose DN with AUCs of 0.897, 0.624, and 0.983, respectively. A negative correlation with eGFR in R of −0.658, −0.176, and −0.628, respectively, in the GSE30528 dataset. Zhou et al. (4) identified CAV1, COL1A2, VWF, FN1, and ITGB2 as having an advantage in assessing DN with an AUC >0.8. Many other studies also screened a series of biomarkers that increased expression in DN compared to controls, and there was certain relevance between biomarkers and clinical parameters like eGFR, ACR, and so on (34–37). All the findings lacked experimental validation and correlation analysis with immunity.

In this article, three DN expression profile datasets from GEO were downloaded and merged. After removing the batch effect, 509 DEGs were obtained with a cut-off standard at |log2FC| >0.5 and adjusted P <0.05. According to the results of functional analysis, both GO and KEGG analysis were tied to the immune system, such as “leukocyte cell–cell adhesion,” “T-cell activation,” “MHC protein complex,” “MHC class II protein complex,” “integrin binding,” “chemokine receptor binding,” “rheumatoid arthritis,” “chemokine signaling pathway,” “antigen processing and presentation,” and so on, suggesting a high correlation between the development of renal tubular injury in DN and the infiltration differential of immune cells. Following this, a GSEA algorithm using an KEGG-annotated gene set based on 37 controls and 45 DN sample expression profiles was performed to further identify the key pathways. After comparing with KEGG-DEGs, the two crossed pathways, namely “chemokine signaling pathway” and “cell adhesion molecules,” were determined, including 36 intersected genes with an obvious positive correlation. Finally, based on PPI network analysis and strict screening using two independent methods, LASSO regression and ROC, three core genes, CCR2, CX3CR1, and SELP, were identified as biomarkers with efficient diagnostic capability for DN, which also included the above 36 intersected genes from the two crossed pathways. No matter the training data or validated data, there were high AUC, sensitivity, and specificity in the diagnosis of DN in both the three independent factors and their combination. Amazingly and surprisingly, it merits our attention that the AUC of the combination of CCR2, CX3CR1, and SELP could reach sensitivity = 100%, and specificity = 100.00% in both merged training datasets, GSE30529–GSE99325–GSE104954, two independent validated datasets, GSE175759 and GSE47184, and IHC detection of biopsy tissues.

CCR2, namely C–C motif chemokine receptor 2, located on chromosome 3, is a member of the G protein-coupled receptor (GPCR) superfamily and a receptor of monocyte chemoattractant proteins (MCP) 1–4, which are chemical inducers of proinflammatory response (38). CCR2 exists on the surface of a variety of immune cells and can guide immune cells to reach inflammatory and tumor sites. By connecting with ligands, including MCP-1, CCR2 recruits the movement and activation of inflammatory cells. As is well known, MCP-1, the main ligand of CCR2 and named CCL2, has emerged as a very vital regulator of DN and has an increasing expression in the renal tissues of diabetic animals (39). There was strong evidence that MCP-1 is significantly upregulated and positively correlated with the degree of tubulointerstitial injury in patients with DN, suggesting that MCP-1 may be involved in the development process of DN and could be a potential diagnostic marker (40–42). As the major receptor of MCP-1, CCR2-expressing macrophages promote renal injury and fibrosis in DN (43). Furthermore, the knockout of CCR2 could reduce the incidence of glomerulosclerosis and secondary tubulointerstitial damage (43). In diabetic db/db mice, inhibiting CCR2 using a small-molecule antagonist can alleviate proteinuria, glomerulosclerosis, and kidney failure (44). Prominently, blocking the CCL2/CCR2 pathway in diabetics and targeting CCR2 have been potential therapeutic interventions and hot topics to limit progressive renal injury. Awad et al. (45) showed that both pharmacological blockade and genetic deficiency of CCR2 could alleviate renal tissue injury in diabetic mice by reducing albuminuria, blood urea nitrogen (BUN), plasma creatinine, histological changes, kidney fibronectin expression, macrophage recruitment, and inflammatory cytokine production in Ins2Akita and STZ-induced diabetic kidney disease. Du et al. (46) found that DN kidney damage could be mitigated by inhibiting macrophage infiltration and downregulating the MCP-1/CCR2 signaling pathway in DN. In addition, two kinds of CCR2 antagonism, rs504393 and ro5234444, could block the development of DN by decreasing macrophage infiltration of the kidney in type 2 diabetes mice (44, 47). A multicenter, randomized trial conducted by de Zeeuw’s team showed that compared to 111 DN patients treated by placebo, 221 patients with DN had a secondary decline in albuminuria given CCX140-B, a selective inhibitor of CCR2, based on standard care with angiotensin-converting enzyme (ACE) inhibitors or angiotensin receptor blockers (ARBs) (48). In our article, CCR2 was upregulated in renal tubular tissues of DN than controls and has a high effective diagnostic ability for DN (AUC = 0.859, sensitivity = 77.78%, specificity = 94.59% in a merged dataset; AUC = 0.939, sensitivity = 100.00%, specificity = 90.91%; and AUC = 0.931, sensitivity = 88.89%, specificity = 100.00% in two validation datasets, respectively, GSE175759 and GSE47184; AUC = 0.958, sensitivity = 92.5%, specificity = 96.7% in IHC validation).

CX3CR1, C-X3-C motif chemokine receptor 1, is a specific membrane-bound receptor of fractalkine (CX3CL1) and belongs to the chemokine receptor superfamily. Currently, CX3CR1 is expressed on the membranes of natural killer cells (NK cells), tubular cells, mast cells, platelets, dendritic cells (DCs), effector T cells, renal cancer cells, vascular smooth cells, mesenchymal cells, and monocytes/macrophages (49, 50). Resembling CCR2, it has seven transmembrane G-protein coupled domains, and it is close to the CCR gene family; it is located at 3p21-3pter (51). Both CX3CR1 and its exclusive ligand, CX3CL1, were upregulated in the kidney in the DN group (50, 52, 53). Accompanying CX3CL1, which is mainly located in the renal tubular epithelium, especially in inflammatory kidney tissues, CX3CR1+ T cells and monocytes are ubiquitously expressed in renal tissues with inflammation in patients (54, 55). Kikuchi et al. (56) tested that CX3CR1 mRNA expression was increased in STZ-diabetic rats at 4 weeks, and the distribution of CX3CR1-positive cells in diabetic glomeruli was also raised at 8 weeks. Moreover, the upregulation of fractalkine and CX3CR1 in the early stages of DN suggested that they may play a crucial role in the progression of DN (56). Furthermore, Song and his colleague showed that there were no obvious changes in plasma glucose level in diabetic CX3CR1^−/−^ mice, while the decline in markers of renal inflammation fibrosis and ECM, such as collagen, fractional mesangial area, and fibronectin, was markedly observed compared with diabetic WT mice (57). Proverbially, the CX3CL1/CX3CR1 axis is significantly related to anti-inflammatory, anti-fibrosis, anti-rejection, and anti-cancer activities in the treatment of renal diseases. Once activation of the CX3CL1/CX3CR1 axis by their combination occurs, a cascade through multiple signaling pathways in the kidney system is initiated, including ROS/MAPKs, Raf/MEK1/2-ERK1/2-AKT/PI3K, and NF-κB. The CX3CL1/CX3CR1 axis directly upregulates the expansion of mesangial cells in diabetes nephropathy through ROS and MAPK (58). So far, no study has focused on the biomarker CX3CR1 in DN. In this study, the results reveal that CX3CR1 expression may be a promising and valuable diagnostic efficiency hallmark in kidney tissues of DN patients with high diagnostic efficacy at AUC = 0.921, sensitivity = 82.22%, specificity = 97.30% in a merged dataset, AUC = 0.939, sensitivity = 100.00%, specificity = 90.91%, and AUC = 0.917, sensitivity = 83.33%, specificity = 100.00% in two validation datasets, respectively, GSE175759 and GSE47184, and AUC = 0.993, sensitivity = 97.5%, specificity = 100.0% in experimental validation.

SELP, also named CD62 or P-selectin, is a kind of glycoprotein and the largest of the selectins with 140 kDa, stored in the α-granules of platelets and in the Weibel–Palade bodies of endothelial cells, and functions on leukocyte recruitment, leukocyte rolling, and platelet adhesion (59). Functionally, as part of the role of cell adhesion, P-selectin could promptly move to the plasma membrane, interacting with its ligands during inflammation (60). Structurally, P-selectin is composed of an extracellular region with an N-terminal lectin domain, an epidermal growth factor motif (EGF), and specifically nine regulatory protein repeats (SCRs), a transmembrane section, and a short intracytoplasmic tail (60, 61). The relationship between P-selectin and DN has been reported by some scholars. A study reported that P-selectin in biopsy kidney tissue of patients with DN was higher than in other glomerular diseases (62). Wang et al. (63) found that the expression level of plasma P-selectin in patients with type 2 diabetes was raised, and with DN development, accompanied by the progressive elevation of plasma p-selectin, the highest expression levels existed in patients with significant renal insufficiency, suggesting a positive correlation between P-selectin and the severity of DN. Bavbek’s team also found higher plasma levels of P-selectin in DM patients compared with controls and in DM patients with proteinuria than without proteinuria (64). Another study reported that P-selectin expression in DN may be induced by NF-κB activation through P50 to participate in the pathogenesis of DN (65). Like the above studies, our research also found a higher expression of SELP in DN than in the control, and the diagnostic value of SELP was assessed by ROC, which showed high AUC, sensitivity, and specificity both in the training dataset, two validated datasets, and biopsy tissue validation.

Proverbially, inflammatory processes with immune modulation are dramatically involved in both the development and progression of structural deterioration in DN. There is undoubtedly evidence that inflammatory cell recruitment, infiltration, and activation play a crucial role in the development and progression of DN. Once released by scathed kidney cells, the inflammatory remodeling progress would be triggered, and the DN progression would be mediated by those lesion or danger signals by initiating immune cells. Growing evidence was reported that pro-inflammatory chemokines, cytokines, growth factors, adhesion molecules, nuclear factors, as well as immune cells, play a major role in the pathogenesis of DN and its complications. Infiltration of immune cells, including lymphocyte cells, macrophages, monocyte cells, and mast cells, into the kidney has been reported. A large amount of evidence supports that the inflammatory components of the tubulointerstitium, especially the proximal tubular epithelial cells, play a central role in the pathogenesis of DN (66, 67).

Generally, the present results highly confirm those previous studies. In this study, kidney tissues in DN had a broader and higher infiltration of immune-related cells and types in comparison with control tissues. Interestingly, it showed general positive correlations among these immune signatures, especially for type I IFN response, MHC class I, cytolytic activity, type II IFN response, pDCs, T-cell co-stimulation, HLA, inflammation-promoting, parainflammation, TIL, CCR, and checkpoint, among which highly positive correlations existed. Moreover, 20 kinds of 29 immune signatures had significant differential distributions based on the Wilcoxon rest. More accurately, consequently, based on the intersection of Wilcoxon test and LASSO regression, seven immune-related cells and types, namely APC co-stimulation, CD8+ T cells, checkpoint, cytolytic activity, macrophages, MHC class I, and parainflammation in the DN group, exhibited a marked infiltration advantage.

Noteworthily, the upregulated infiltration of macrophages in DN had been found from both animal models and kidney biopsy specimens of DN patients. It was reported that macrophage accumulation had been found in both glomeruli and interstitium (68–70). The quantity of macrophages in the interstitium is in direct proportion to the proteinuria level in the STZ model of type I diabetes (69). Once recruited to the kidney, macrophages have been proposed to mediate renal injury through a variety of mechanisms, including the production of reactive oxygen species (ROS), cytokines, and proteases, which lead to tissue damage and ultimately to fibrosis (71). Gradually, studies have shown that the expression of ICAM-1 and MCP-1 in renal tubular cells was elevated due to high blood glucose levels and stimulation of advanced glycation end products, and then infiltration of macrophages followed. Infiltrating macrophages mediate renal injury by releasing lysosomal enzymes, nitric oxide, ROS, transforming growth factor, vascular endothelial growth factor, and cytokines (72, 73). Moreover, the accumulation of macrophages in DN indicates the decline of renal function, followed by inflammation progression in DN induced by the macrophage-derived products. As a result, there was a close connection between macrophage accumulation and the development of renal lesions and the decline of renal function (70, 74). Besides, a growing number of studies have reported that targeting CCR2, one of three selected biomarkers in our study, could relieve macrophage infiltration and ameliorate inflammation to inhibit DN progression (44–47). It is consistent with our results that CCR2 was significantly positive in macrophages in DN. As for T cells, recent studies have suggested a momentous role for T-cell recruitment into kidney tissue, accompanied by the recruitment of macrophages, in diabetic nephropathy (75). Higher accumulations of CD4+ and CD8+ T cells had been detected in the glomeruli of diabetic NOD mice than controls (76). Moon (77) reported an observed increase in CD4+, CD8+, and CD20+ cells in renal interstitial tissues of Type II diabetic patients and close links between CD4+ and CD20+ cells and proteinuria, indicating the underlying immunopathological correlations in DN with disorderly infiltration and the activation of T cells in renal interstitial tissues. Another study found higher infiltration of CD4+T cells, CD8 T cells, and macrophages in the kidney tissues of STZ-induced diabetic rats and significantly higher expression of CD4, CD8, MHC classes I and II, and the proinflammatory cytokines tumor necrosis factor-a, interferon-γ, and nitric oxide (NO) in diabetic kidneys in comparison with control (69). Notably, CD8+ T cells, the subcategory of leukocytes, have a strong pro-inflammatory effect and are involved in mediating immunity by direct cell–cell signaling *via* surface molecules and indirect signaling *via* cytokines in kidney damage. It is markedly elevated in DN (69, 76–78) and has gradually become a potential therapeutic target of DN (78, 79). Zhang et al. (62) exhibited the therapeutic value of mesenchymal stem cells by suppressing CD8+ T-cell proliferation and activation mediated by CD103+ DCs in DN rats. Seo (79) and his colleagues reported that Mycophenolate Mofetil can alleviate diabetic nephropathy in db/db mice, followed by decreased albuminuria, attenuated mesangial expansion, and profibrotic mRNA expressions through downregulating the infiltrated CD4+ and CD8+ T cells. Besides, as cytotoxic T lymphocytes, CD8+ T cells might be responsible for the kidney damage in DN. After the secretion of cytokines, CD8+T cells can be recruited to the inflammatory location by interacting with MHC class I antigen, which is commonly expressed on all nucleated cells (69, 80). It can be a logical explanation for our study that CD8+ T cells and MHC class I antigen were coincidentally elevated in the DN group. Moreover, dendritic cells, HLA, neutrophils, Th1 cells, Th2 cells, and so on, were demonstrated to play a crucial role in the development and process of DN (11, 76, 81–84). In view of this, our research is consistent with previous reports and highlights the importance of those immune-related cells and types in the pathogenesis of DN through bioinformatic analysis.

Given the pivotal role of immune infiltrating cells and biomarkers in DN, the relationships between three biomarkers and seven significant immune signatures were analyzed further by the Spearman algorithm. Meaningfully, three biomarkers are highly and positively correlated with these immune-related cells and types, which is highly consistent with the crucial role of pro-inflammatory factors and immune-related cells and types in kidney damage in patients with DN. Collectively, all of these findings provide logical ideas about how the immune system modulates in DN. This may lead to the discovery of earlier and more reliable biomarkers and, hopefully, the identification of new therapeutic targets in diabetic kidney disease.

Besides, impossible therapeutic compounds were also explored using CMap, an online tool analyzing underlying drugs based on the 36 intersection genes in two intersected pathways between KEGG-DEGs and GSEA-KEGG analysis in this article. The CMap database (https://clue.io/) is a gene expression database built by researchers from Harvard, Cambridge University, and the Massachusetts Institute of Technology. It is a biological application database related to distractors, gene expression, and diseases that was established based on gene expression differences using different distractors (including small molecules) to deal with human cells (4). According to the correlation between genes, diseases, and drugs established by gene expression profiles, it is helpful for researchers to quickly use gene expression profile data to compare drugs highly related to diseases, infer the main structure of most drug molecules, and summarize the possible mechanism of action of drug molecules in the field of drug research and development. In this research, tetrabenazine, dilazep, tomelukast, KIN001-220, azacytidine, umbelliferone, lysylphenylalanyl-tyrosine, memantine, phensuximide, and BIBU-1361 were the top 10 compounds with negative correlations, which may reverse the alterations. Specifically, dilazep, as an antiplatelet drug, is a kind of adenosine reuptake inhibitor, calcium channel antagonist, and platelet aggregation inhibitor. It has a vasodilator effect, and it can selectively expand the coronary arteries and increase coronary blood flow. It has been reported that dilazep could improve kidney function. Nakazawa et al. (85) reported that dilazep dihydrochloride could significantly suppress glomerulosclerosis and glomerular adhesion to Bowman’s capsules in rats with Masugi nephritis. Dilazep dihydrochloride was also found to improve proteinuria in patients with DN (86–88), which suggested that platelet activation played a pivotal role in the development and process of DN (89). Another study reported that dilazep may be useful in preventing renal deterioration in the early stages of type II DN (90). In a multicenter study that was researched the clinical efficacy of dilazep dihydrochloride in the microalbuminuria stage of DN, 37 DN patients with microalbuminuria were given orally 300 mg/day of dilazep dihydrochloride. Compared with before, the mean albuminuria was noticeably lower, and the urinary NAG activity improved after treatment with the drug. Meanwhile, no renal function damage was found at this stage. It appears that early administration of dilazep dihydrochloride may contribute to improving proteinuria and preventing renal dysfunction in patients with DN (86). In Ebihara’s study, 22 patients with IgA nephropathy and 20 healthy controls were recruited, and among them, 14 patients in stage II or III were treated with dilazep dihydrochloride. In the study, the P-selectin expression level in plasma and urine in patients with IgA nephropathy was detected, and the relationship between the patients’ histology and urinary protein excretion was analyzed. Therefore, plasma P-selectin is a helpful biomarker for the activity of IgA nephropathy, and dilazep dihydrochloride is an efficacious drug for reducing plasma soluble P-selectin levels in patients with IgA nephropathy (91). Interestingly and coincidentally, P-selectin, also named SELP, as a marker representing disease activity, cellular activation, and inflammatory mediators, is one of three selected biomarkers related to DN in our study. Dilazep is a potential therapeutic agent for the treatment of DN patients, as analyzed by CMap in our study. The high similarity with Ebihara’s study confirms the reliability and accuracy of the results, which suggest that P-selectin is a very promising biomarker for DN and that dilazep is a prospective drug to improve renal function in DN with the decline of p-selectin.

## 5 Conclusion

Conclusively, the present article identified three core and prospective biomarke<rs implicated in diabetic tubulointerstitial lesions, which had close links with immune cells and types and would be a future underlying target for the diagnosis and immunotherapy of DN. Besides, dilazep, a small molecular agent, was found to be promising therapeutic drug in diabetic renal disease. However, the present study also had some limitations. As biomarkers, plasma levels detection in clinic and deeper basic mechanism studies *in vitro* and *in vivo* are needed to validate the feasibility of transformation applied to diabetic tubule lesions. Most importantly, the analysis of the relationship between immune cell infiltration and diabetes tubulointerstitial injury provides a novel potential approach and strategy for immunotherapy to improve diabetic tubulointerstitial injury in DN patients.

## Data availability statement

The original contributions presented in the study are included in the article/**Supplementary Material**. Further inquiries can be directed to the corresponding author.

## Ethics statement

The studies involving human participants were reviewed and approved by The Ethics Committee of Second Hospital of Hebei Medical University. The patients/participants provided their written informed consent to participate in this study.

## Author contributions

YS and HZ designed the research and collected the data. HZ analyzed the data and wrote the paper. HZ, LM, and ZY helped interpreted the data. HZ and LM prepared all figures and tables. LM and ZY revised the language of the article. All authors listed have made a substantial, direct, and intellectual contribution to the work and approved it for publication.
