# Supplementary material for: Identification of a novel immune landscape signature as effective diagnostic markers related to immune cell infiltration in diabetic nephropathy
Source: Front Immunol. 2023 Mar 8;14:1113212. doi: 10.3389/fimmu.2023.1113212 (PMC10030848; doi:10.3389/fimmu.2023.1113212)
Supplement: Supplementary file 1 [file DataSheet_1.docx]

Supplementary Material

# Supplementary Figures and Tables

## Supplementary Table 1

## Table S1 The information of datasets

| Datasets | Platform | Tissue | Sample | | Experiment | Attribute |
| --- | --- | --- | --- | --- | --- | --- |
|  |  | Homo sapiens | Control | DN | type |  |
| GSE30529 | GPL571(Human Genome U133A 2.0 Array) | tubulointerstitium | 12 | 10 | array | Test |
| GSE99325 | GPL19184(Human Genome U133A Array); GPL19109(Human Genome U133 Plus 2.0 Array) | tubulointerstitium | 4 | 18 | array | Test |
| GSE104954 | GPL24120(Human Genome U133A Array); GPL22945(Human Genome U133 Plus 2.0 Array) | tubulointerstitium | 21 | 17 | array | Test |
| GSE47184 | GPL14663;( Human Genome HG‐U133A Custom CDF) GPL11670(Human Genome U133 Plus 2.0 Array) | tubulointerstitium | 4 | 28 | array | Validation |
| GSE175759 | GPL16791(Illumina HiSeq 2500) | tubulointerstitium | 22 | 3 | RNA-seq | Validation |

##
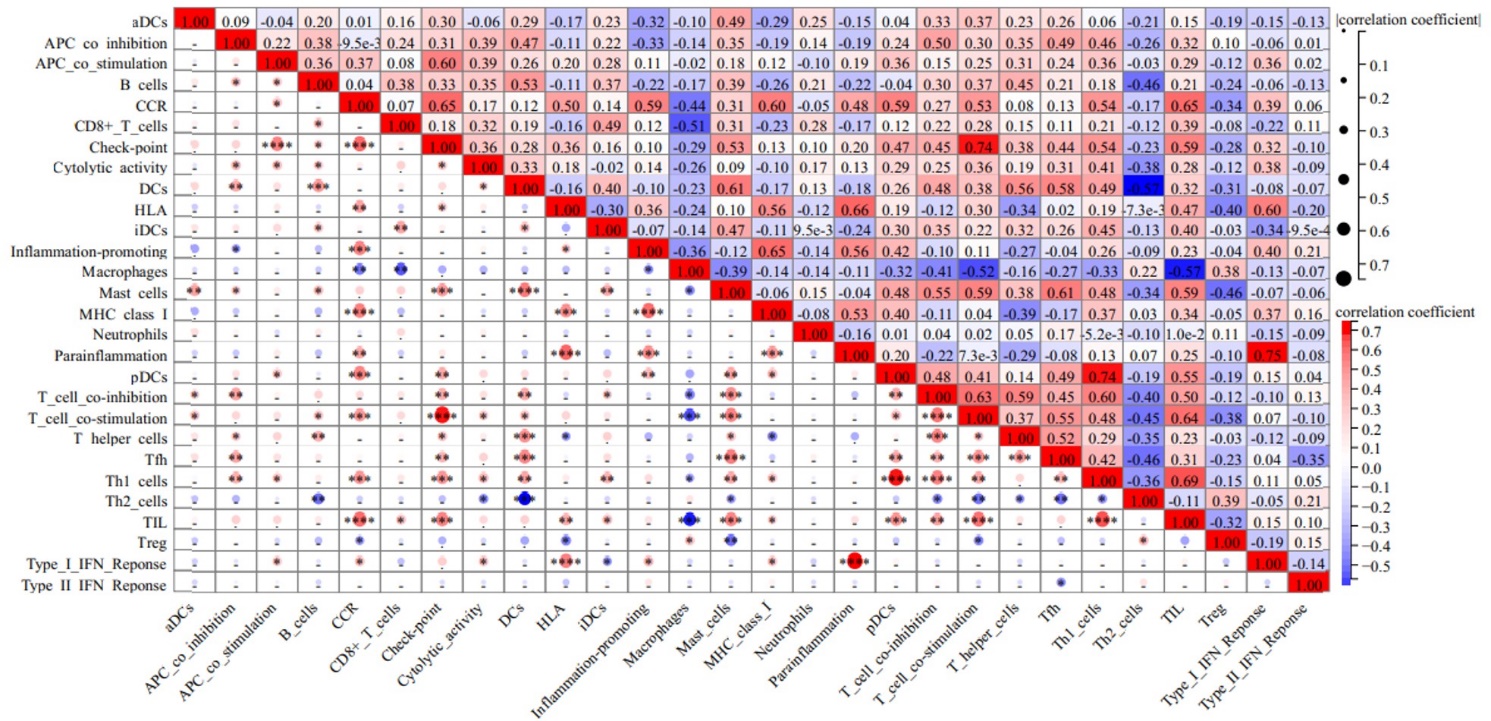
Supplementary Figure 1

## Figure S1. The Correlation analyses among the immune signatures calculated by ssGSEA in control group.

Red: positive correlation; blue: negative correlation.

** P < 0.05, ** P < 0.01, *** P < 0.001, **** P<0.0001*
